# Syphilis in pregnancy and congenital syphilis: women’s experiences from the perspective of symbolic interactionism

**DOI:** 10.1590/0034-7167-2022-0210

**Published:** 2022-11-28

**Authors:** Jéssica Batistela Vicente, Gabriel Zanin Sanguino, Maria Regina Pontes Luz Riccioppo, Maiara Rodrigues dos Santos, Maria Cândida de Carvalho Furtado

**Affiliations:** I Universidade de São Paulo. São Paulo, São Paulo, Brazil; II Universidade de São Paulo. Ribeirão Preto, São Paulo, Brazil

**Keywords:** Pregnancy, Syphilis, Syphilis, Congenital, Child Care, Symbolic Interactionism, Embarazo, Sífilis, Sífilis Congénita, Cuidado del Niño, Interaccionismo Simbólico, Gravidez, Sífilis, Sífilis Congênita, Cuidado da Criança, Interacionismo Simbólico

## Abstract

**Objectives:**

to understand the meanings attributed by women to the diagnosis and treatment of syphilis and congenital syphilis, and to outpatient follow-up of their children.

**Methods:**

this is a qualitative study conducted with 30 mothers of children with congenital syphilis using audio-recorded semi-structured interviews, which were submitted to inductive thematic analysis. Symbolic interactionism was the theoretical framework considered in this study.

**Results:**

two themes were identified, showing the maternal diagnosis involved shock, guilt, and fear of social exclusion, in addition to frustration due to failure to prevent vertical transmission. Moreover, the painful clinical procedures for the child’s treatment enhanced maternal guilt, and the symbolic process of re-signification of the disease/treatment took place with the child’s healing.

**Final Considerations:**

understanding the intersubjective aspects involved in this experience helps nurses rethink their care practice and contributes to their critical role in the context of syphilis.

## RESUMO

**Objetivos:**

compreender o significado atribuído pelas mulheres ao diagnóstico de sífilis e sífilis congênita, ao tratamento e acompanhamento ambulatorial da criança.

**Métodos:**

estudo qualitativo conduzido com 30 mães de crianças com sífilis congênita, por meio de entrevistas semiestruturadas, audiogravadas e submetidas a Análise Temática Indutiva. Adotou-se, por referencial teórico, o Interacionismo Simbólico.

**Resultados:**

dois temas emergiram e revelaram que o diagnóstico materno foi permeado por choque, culpa e medo da exclusão social, além de frustração ante o insucesso em evitar a transmissão vertical. Ainda, os procedimentos clínicos dolorosos para tratamento da criança potencializaram a culpa materna, e o processo simbólico de ressignificação da doença/tratamento aconteceu com a cura da criança.

**Considerações Finais:**

a compreensão dos aspectos intersubjetivos envolvidos nessa experiência instrumentaliza o enfermeiro para repensar sua prática assistencial e contribui para seu maior protagonismo no contexto da sífilis.

## RESUMEN

**Objetivos:**

comprender el significado atribuido por las mujeres al diagnóstico de sífilis y sífilis congénita, al tratamiento y seguimiento ambulatorio del niño.

**Métodos:**

estudio cualitativo realizado con 30 madres de niños con sífilis congénita mediante entrevistas semiestructuradas, grabadas en audio y sometidas a Análisis Temático Inductivo. Se adoptó como referencial teórico el Interaccionismo Simbólico.

**Resultados:**

surgieron dos temas revelando que el diagnóstico materno fue teñido por shock, culpa y miedo a la exclusión social, además de frustración frente al fracaso que no permitió evitar la transmisión vertical. También los procedimientos clínicos dolorosos para el tratamiento de los niños potenciaron la culpa maternal, y el proceso simbólico de resignificación de la enfermedad/tratamiento se dio con la cura del niño.

**Consideraciones Finales:**

la comprensión de los aspectos intersubjetivos involucrados en esta experiencia instrumentan al enfermero para reconsiderar su práctica asistencial y contribuye a un mayor protagonismo en el contexto de la sífilis.

## INTRODUCTION

Syphilis in pregnant women is associated with risks for both the woman and the fetus due to vertical transmission (congenital syphilis), such as abortion, premature birth, neonatal death, low birth weight, neonatal neurological impairment, including cognitive delay, vision loss, convulsive disorders, and bone malformations^(1-3)^. Congenital syphilis (CS) is a condition that can be prevented by controlling syphilis in women and their sexual partners during family or prenatal planning^(3-5)^.

In 2016, the worldwide prevalence of syphilis in pregnant women was estimated at 0.69%, resulting in an overall CS rate of 473 cases per 100,000 live births^(1)^. In Brazil, a considerable increase has been observed in the CS rate - in 2008, 2 cases were reported per 1,000 live births and in 2020, 7.7 cases per 1,000 live births^(6)^, very far from the goal of 0.5 cases per 1,000 live births by 2015, as defined by the World Health Organization (WHO), also confirmed for 2030^(7)^.

The prevention of vertical transmission of syphilis supports the Sustainable Development Goals (SDGs) issued by the United Nations (UN) for the period from 2016 to 2030, to which Brazil is a signatory. One of the targets is to end preventable deaths of newborns (NB) and children under five years old^(8)^. Worldwide, the neonatal component corresponds to 44% of infant mortality^(9)^ - the main causes are prematurity and congenital malformation^(10)^, which can be consequences of CS^(1)^.

Congenital syphilis is considered a marker of prenatal care quality, since vertical transmission can be 100% prevented with timely diagnosis and adequate treatment of the pregnant woman^(3,5)^. Prevention and treatment involve complex issues related to gender, sexuality, marital infidelity, prejudice, and discrimination within the family and in health services^(11)^.

The experience of being diagnosed with syphilis and transmitting the disease to the child generates feelings of sadness, fear, shock, guilt^(12-18)^ that become more intense as the NB remains hospitalized to treat the disease^(13,15-17)^. Stigma by health professionals and family members is also observed in the diagnosis of syphilis^(12-13,15-17)^, leading to omission of the disease within the family^(12-13,15-17)^, which restricts support at an important moment for building parenthood^(12)^.

Nurses can prevent vertical transmission through health promotion and self-care for women and their partners, as well as quality prenatal care^(3,5,12,19-20)^. In the hospital environment, when CS is diagnosed, many mothers are surprised and shocked, as they did not expect the child to be infected and need support and guidance from nurses regarding the diagnosis, treatment, and health care, not only for them, but also with the NB^(13,15-16,18)^.

Although many studies have been developed with a focus on prenatal care and treatments for both syphilis during pregnancy and CS, the evidence is based on epidemiological data^(1-4,21-23)^. There is a gap in the literature about the symbolic representations and meanings involved in actions and practices of women before the child’s birth to avoid vertical transmission and, after birth, when following up the health of the child with CS. Understanding the intersubjective aspects involved in this experience can support and enhance nursing care. In this sense, the guiding question of this study was: What are the meanings attributed by women to the diagnosis and treatment of syphilis and CS, and to outpatient follow-up of their children?

## OBJECTIVES

To understand the meanings attributed by women to the diagnosis and treatment of syphilis and CS, and to outpatient follow-up of their children.

## METHODS

### Ethical aspects

This study was approved by the Research Ethics Committee of the Ribeirão Preto College of Nursing at the University of São Paulo and complied with the ethical requirements in Resolution 466/2012 of the National Health Council. Participants were identified by the letter E, followed by an Arabic numeral.

### Theoretical and methodological framework

The theoretical framework used in this study was symbolic interactionism (SI), since it values the meaning of the phenomenon for individuals in a given context, aiming to understand the deeper dimension of their actions and interactions in the search for these meanings. From the SI perspective, things (objects, human actions, institutions, ideas, activities of others in everyday life) assume meanings in the individual’s interaction with others and while the individual consciously watches, reflects and thinks about the object and then interprets it^(24)^.

Symbolic interactionism helps understand the meanings of diagnosis, treatment, and outpatient follow-up of the children’s health that emerge from interactions, and defines the actions of mothers in the child care process. The symbol (CS), the self (self-awareness), the mind (where a subject understands the other), human actions (active decision-making process), and interactions (communication and interpretation) support these women while they build their trajectories.

### Study design

This is a qualitative study with an approach based on SI^(24)^ and inductive thematic analysis^(25)^. In order to qualify the study production, the guidelines of the Consolidated Criteria for Reporting Qualitative Research (COREQ)^(26)^ were adopted.

### Study setting

This study was conducted in a city in the northeast region of the state of São Paulo, which has a broad health network offering primary care services, high complexity and specialized care. In 2014, a reference center for specialties was inaugurated, which includes a municipal outpatient service for congenital infections, where our study was conducted. The reference center treats patients from the Brazilian Unified Health System (SUS) living in the municipality, with sexually transmitted infections (STIs), tuberculosis, and viral hepatitis. It has a specialized care service and a testing and reception center. The reference center is linked to municipal programs for primary care, women’s health care, and child and adolescent health care. For newborns with CS, referral is made by the *Floresce uma Vida* program team, under the child and adolescent health care program. The team schedules the first appointment of the NB in primary care after discharge from the maternity ward. If risks are identified for the neonate, visits are also scheduled in specialized services such as the early stimulation service (newborns with development risk) and the municipal outpatient service for congenital infections (newborns with CS or who are exposed to maternal syphilis in maternity hospitals). At the outpatient service, children are followed up with monthly visits up to 6 months of age, and bimonthly after that period.

### Data source

Thirty mothers of children with CS were contacted and all of them agreed to participate in the study. Inclusion criteria were: mothers aged 18 years or older and children being followed up by the outpatient service for at least three months (follow-up appointments). This follow-up period was selected as it would constitute an important limiting factor to understand this experience. Mothers were approached in the outpatient waiting room, on the days of child care, and were invited to participate in the study.

### Data collection and organization

Data collection took place between March and October 2018 through individual semi-structured interviews conducted by the main researcher with experience in qualitative studies, in a private room provided by the health service. The interviews were audio-recorded and lasted around 40 minutes.

The interview was based on the guiding request: “Tell me about your child’s care, from birth to this moment”. Supporting questions sought to encourage mothers to tell their experience in detail, for instance: “How and when did you find out you had syphilis?”; “Did you know about the effects of this disease on your child?”; “When and how did you find out your child had CS?”; “How was the treatment (hospitalization period)?”; “How is the outpatient follow-up going?”; “What are your perceptions and feelings regarding CS?”.

A field diary was filled with relevant notes to the interview process, which cannot be audio recorded (use of non-verbal language such as gestures, postures, and facial expressions). This information was included in interview transcripts. The field diary was filled at the end of each interview. All participants were able to listen to their interviews, making further comments or corrections; however, no participant requested any insertion or adaptation.

Data collection ended when the researcher reached the study objective. An ideal qualitative sample reflects the multiple dimensions of the phenomenon and seeks quality of actions and interactions, highlighting the researcher’s certainty of finding the internal logic of the study objective^(27)^.

### Data analysis

Interviews were transcribed by the main researcher and validated by another researcher. Through inductive thematic analysis^(25)^, the latent content analysis was performed, guided by data and interpreted according to SI. Inductive thematic analysis goes beyond the semantic content of data and identifies ideas, assumptions, and concepts, while the development of themes involves interpretation, not just data description^(25)^. This analysis consists of six stages. In stage 1, the repeated reading of data was performed, seeking meanings and patterns; in stage 2, the initial codes were organized; in stage 3, themes were identified by highlighting repeated words, expressions, and phrases and the contents addressed with more emphasis, then an initial thematic map was built. Themes were refined in stage 4, when the thematic map was developed. In stage 5, the themes were defined and named (final thematic map). Also in stage 5, data analysis was written, with the organization of excerpts in agreement with the supporting text and interpretation based on SI; and in stage 6, representative data excerpts were defined for each theme, and the analysis, study question, literature, and theoretical framework were all correlated. Two themes were identified after the analysis: ‘Diagnosis of syphilis and transmission of the disease to children: women’s experience from shock to frustration’ and ‘Resignification of the experience with the disease: treatment and outpatient follow-up of the children’.

## RESULTS

The study participants were between 18 and 38 years old (mean age 28 years); 21 of them were married, 7 were divorced and 2 were single; 10 were multiparous (average of 2 children). Regarding the children, 16 were male and 14 were female, aged 3 to 12 months (average of 5 months).

### Theme 1: Diagnosis of syphilis and transmission of the disease to children: women’s experience from shock to frustration

When the pregnant women were diagnosed with syphilis during pregnancy, they related the disease to the human immunodeficiency virus (HIV), an incurable STI, they felt shocked, ashamed, guilty, and worried about transmitting the disease to their children. The symbolic meaning of syphilis as a disease related to promiscuity was aggravated by the fear of facing stigmatized social attitudes, especially from family members and health professionals, so they opted for confidentiality of disease information to protect themselves from prejudice and judgment.

*No, nobody knows in my family, I chose not to tell anyone* [...] *I feel partially ashamed to explain, they would want to know how it happened, oh how did you get that? Not even my mother, my sister, nobody knows, just me and my husband.* (E13)

*Because my father is like that, I had a cousin who died of HIV, and he* [father] *used to say, oh, he kept despising my cousin, and he said that when someone has it, you can get it from contact, so he mistreated him a lot. Then my mother said, don’t tell anything to your father* [...]. (E8)

*I was very afraid to tell people because when people are uninformed and don’t understand anything, it’s like my mother in this sense, because my mother is very ignorant, she’s like that, if you tell her that a person has AIDS, she doesn’t even sit where the person sat because she’s afraid of transmission* [...]. (E15)

This symbolic meaning was rebuilt as they acquired more knowledge about the disease, maternal treatment, healing, and prevention of vertical transmission. These women, as social actors, assumed the role of mothers, and made an effort to protect their children. Such effort represents their attitudes during treatment, when they had feelings of concern, guilt, and responsibility for the well-being of their babies.

*So, during her prenatal care* [referring to the child] *I found out that I had syphilis, I was two months pregnant, then I did the treatment and we are still following up* [...]. *He* [health center doctor] *said that if I didn’t treat her, she could be born blind, deaf, with some physical problem, so I didn’t think twice about the treatment* [...]. *Wow, it’s a very big responsibility, you get very worried, every ultrasound you do you want to know if everything went really well.* (E7)

*She* [nurse] *explained everything to me, calmed me down, because I was desperate, you know? She explained to me there was a treatment available, and that if I did the treatment correctly, she* [daughter] *would be fine for the rest of her life, so I did it right away.* (E10)

*When I found out I was shocked, I was nervous and he* [health center doctor] *just said it could be transmitted to the baby, and if I didn’t take care of it, the baby would suffer* [...]. *Then I took Benzetacil^®^, I did everything right and she* [daughter] *is doing well*. (E9)

To the diagnosis of CS, the women attributed the meaning of failure in their parental role, since they made an effort during pregnancy, in vain, to avoid vertical transmission. Guilt, concern, and fear were accentuated by the feeling of frustration due to the failure to prevent CS.

*I was very scared and worried and prayed that nothing serious would happen to him* [...]. (E4)

*I felt very bad, I was really worried, because if I have that, that’s ok, but it is not her* [daughter] *fault, right?* (E19)

*I felt guilty, because I had follow-up care so nothing would happen to her, so she would be born well, and in the end, it was useless. So, I felt guilty for her having this disease, you know?* [...] *like it or not, it was my fault*. (E10)

### Theme 2: Resignification of the experience with the disease: treatment and outpatient follow-up of the children

The children of the study participants were hospitalized for an average of 10 days after birth to be treated for CS with intravenous crystalline penicillin. Seeing their children undergoing a painful clinical procedure, although for healing, increased their guilt and frustration due to vertical transmission.

*I cried, I cried a lot, because I wanted it to be positive for me, not for him, and every time he was going to get an injection* [intravenous medication for treatment]*, I cried a lot* [...] *I woke up at night and dreamed, and prayed, put it on me, but take it off from him. I felt defeated, very defeated, such a tiny little baby!* (E5)

*The day at the maternity hospital, when I saw her* [daughter] *there with the needle receiving* [medication] *I cried a lot, because I felt very guilty*. (E25)

[...] *I lost 12 days, right, I could be at home enjoying my daughter, but we were in the hospital, every 6 hours she received the medication in the vein, then the vein burst and they had to puncture her, it was terrible*. (E28)

Resignification of the experience occurred in the symbolic process of discovering the possible impact of CS on the healthy growth and development of the children, which would be avoided with treatment. Although hospitalization was difficult for the women, it meant healing and the well-being of their babies.

*He was infected and took the injection* [intravenous medication] *because of the infection, to stop it, it was terrible to stay there* [in the hospital], *but good for him, right?* (E12)

*I felt terrible, I just cried. On the first days, you actually feel a lot of strength to stay there, but when you sleep 5 days in the hospital, 6 days in the hospital, you don’t see anything, then you start feeling desperate. We can only hold on for* [pause] [...] *ah I really wanted to go home, but I couldn’t just pick my daughter up and leave, because if I did that, she wouldn’t be treated*. (E28)

After hospital discharge, the children began attending monthly outpatient follow-up appointments for clinical and laboratory evaluation. This follow-up was an opportunity for women to resignify the disease and treatment since prenatal care, and it became a double achievement: the mother and the baby’s healing and the healthy growth and development of the child.

*You have to bring the baby here, right, for me to be informed and see if everything is fine with him, if it will be a problem for his development and growth, and the doctor said no. His two blood tests were negative, mine too; she said that I don’t need to worry because it’s not in his body. Here* [reference center] *I get to understand the disease better* [...]. (E3)

*It was great, the doctor...ah she explained to me everything I wanted to know, she explained most things to me because she had more patience with me* [...]. *Now we are discharged, thank God*. (E10)

*So, since he was born, he’s been treated, so I come here* [reference center]. [...] *thank God the tests were negative, I’ll do the last one to prove it, so thank God, God is giving a victory for me and for him too*. (E12)

*So here* [reference center] *I felt calmer, because the doctor always told me that I didn’t need to be afraid because she was doing very well, and she is, she doesn’t have anything, she is already healed, all the exams were negative*. (E21)

## DISCUSSION

Our study showed the meanings attributed by women to the experience of being diagnosed with syphilis, of vertical transmission of the disease and monitoring the child’s health care, in hospital and outpatient settings. The SI framework helped understand these meanings, as it considers human behavior must be based on the social behavior, which occurs in two dimensions: the overt behavior, which is the observable external behavior, and the covert behavior, which is the internal experience of the individual^(24)^.

For each observable behavior during the child’s health care, there is a covert behavior and, during these behaviors, the social objects of the environment are defined and redefined. In this context, women, as social actors, assumed the role of mothers, which determined their actions and the way they interpreted every moment of this trajectory.

When they were diagnosed with syphilis, the women attributed a meaning to the disease (disease related to promiscuity), and according to SI^(24)^, this interpretation is a result of their social interactions, which change through a process that is constantly developed by these women when faced with situations in their lives.

The diagnosis of an STI in the life of a woman causes fear of different intensities, such as fear of death, fear of transmitting the disease to other people, or fear of social withdrawal^(11)^. In our study, although the women expressed feelings of shock, concern, frustration, fear when the diagnosis was confirmed, and option for confidentiality in agreement with their partners, they did not associate syphilis with death, as they understood that, with proper treatment, there is cure and vertical transmission is prevented.

The treatment of syphilis during pregnancy was interpreted as a way to protect the child and prevent vertical transmission, with feelings of frustration and defeat in case of treatment failure. The prognosis of CS is related to the severity of the intrauterine infection and the period when the woman was treated; thus, early diagnosis in pregnant women and the adoption of adequate therapy prevent vertical transmission, with children being less affected^(28-29)^.

In Brazil, the recommended treatment for syphilis in pregnant women is performed with benzathine benzylpenicillin in an appropriate dose to the clinical stage of the infection, with the first dose given up to 30 days before delivery^(29)^. CS transmission is related to the quality of prenatal care, since vertical transmission can be prevented with quality care, in addition to early diagnosis and proper treatment^(3,18)^.

Several factors contribute to adequate prenatal care, including the gestational age at the beginning of the follow-up, number of visits, and routine exams. Evidence showed that gaps in this care resulted in inadequate or absent treatment in about half of the studied women^(3,21)^. When the diagnosis and treatment of syphilis in pregnant women do not occur early, the consequences for the baby can be serious and lead to premature birth, abortion, stillbirth, and neonatal death^(1-3)^.

In primary care, nurses provide prenatal care to ensure both mother and fetus are healthy during the gestational period and heal infection and prevent vertical transmission. Nurses also talk to the women’s partners, providing guidance and emotional support to both parents, often going beyond clinical issues to fulfill their social, emotional, and psychological needs^(13,19-20)^. Multiprofessional teams must actively seek pregnant women who have not received prenatal care, developing actions to increase awareness of the population about the risks of unprotected sexual practice and the importance of self-care^(3)^.

The possibility of vertical transmission produced constant feelings in these women, such as shock, concern, and responsibility for the child’s health, fear of any sign or symptom in the child, in addition to guilt and frustration. SI explains this phenomenon through social interaction^(24)^, which is the central concept of the theory; then, the perception of the threat of vertical transmission stimulated the interaction of women with social objects and, during the mind activity, they attributed to CS the meaning of a consequence of their behavior and failure in their parental role, which resulted in these feelings.

Similar results from other studies indicated that, when diagnosed with CS, mothers experience sadness, guilt, despair, and frustration in face of their children’s pain^(12-14,17-18)^.

Prolonged hospitalization of the children and the need to undergo painful procedures for exams and treatment increased negative emotions, data that agree with studies that identified feelings of powerlessness, stress, concern, and anxiety related to multiple vein punctures, risk of death, and wait for the results of imaging and laboratory tests^(12-13,15,17)^. Then, nurses can identify such feelings and dedicate time to help these women cope with the disease and minimize suffering^(12,17)^. Nurses ethically committed to comprehensive and humanized care, focused on the family, and not on the disease, can minimize the stigmatization of these women^(12-13)^.

In addition, the children’s hospitalization influences the adaptation of parents, since it is a period of more vulnerability for women, who experience changes and difficult moments of self-care and child care^(12)^. In this context, nurses play an important role in supporting the development of positive parenting by promoting a connection of parents/baby/family and guiding, encouraging and enabling parents to provide responsive care to fulfill the children’s needs^(12)^.

The women’s statements indicate the meanings attributed to their children’s treatment were negative experiences because they had to stay in the hospital longer than they planned and see their children submitted to painful clinical procedures, such as vein puncture for antibiotic therapy. However, guilt and pain caused by the hospitalization were relieved when they understood the disease and the value of such care for the healthy growth and development of their children, in agreement with a study that assessed the parenthood of parents of NB hospitalized with CS^(12)^.

This period in the hospital requires adaptation of routines, including changes in social and work life, and can generate tension and concern due to the time away from home, and away from the family and their other children^(12,30-31)^. With good communication, nurses can ease the anxiety of parents and, consequently, favor the acceptance of the disease, hospitalization, treatment, and help them cope with the situation^(12,30-31)^.

In this context, confidentiality of the diagnosis of syphilis and CS is an important aspect, as it was the strategy found by the study participants to avoid the judgment of other people, including family members, a fact that restricts support at an important moment of building parenthood, also identified in other studies assessing CS^(12-13,15-17)^.

Sexually transmitted infections, such as syphilis, have historically led to social exclusion and prejudice, which can affect the treatment; thus, the role of nurses is essential to demystify the disease^(12-13,15-17,20)^. The stigma of acquiring an STI is associated with cultural factors, considering that, for a long time, STIs were predominant among sex workers, drug users, and homosexuals. Also, it became stronger with HIV infection, which resulted in fear, shame, and rejection, and even today STIs are related to promiscuity and risky behaviors^(11,13)^.

According to data of our study, in addition to omitting the diagnosis, the women associated syphilis with HIV; they attributed to the disease a meaning of something shameful and that should be omitted to avoid the judgment of society and social exclusion. According to SI^(24)^, women are always socially active, observing and identifying situations, mentally creating symbols and interacting with themselves, their families, and the environment. Thus, the situations of prejudice women have witnessed in life, combined with their current experiences, led to such interpretations. Again, nurses play an important role, providing information to resolve doubts, demystify syphilis, and allow its resignification by the women, developing a new way of acting after learning about the disease.

The moment the mothers showed a better understanding of the disease was during outpatient follow-up, with acceptance of the diagnosis, perception of follow-up as essential care for the children and confirmation of healing. The Brazilian Ministry of Health recommends that children with CS should have monthly outpatient follow-up until the sixth month of life, and bimonthly follow-up from the sixth to the 18th month^(29)^. To ensure follow-up of these children, the family must be included in care, meeting their needs and not just the clinical aspects of child care; thus, creating bonds would favor adherence to child follow-up^(32)^.

Despite the evidence reinforcing prenatal care and care after childbirth, the protocols^(29,33)^ tend to focus care on the disease and do not consider the perspective of these women’s experiences, which involve feelings like pain, fear, and doubts, as expressed in the empirical material produced in this study. Provision of care can go beyond this unilateral perspective and integrate actions to listen and understand how women experience the disease, self-care, and care for their children.

Our study found intense intersubjective aspects that these women experienced and resignified at different moments, from diagnosis of the disease to child follow-up in a specialized service. Their perceptions of the disease and how it affects their lives have an impact on their interactions with family, health services, and support networks. Giving voice to these experiences allows learning more about how every individual experienced and interpreted them, thus supporting nursing professionals in providing quality and comprehensive care to these patients.

### Study limitations

Study limitations refer to the meanings attributed to the experience of 30 women, mothers of children with CS. Data presented in this study do not seek generalization or definitive answers, as the subjects are constantly interacting socially, decoding their experiences, and attributing new meanings to their experiences.

### Contributions to nursing

Nurses have - and should assume - an essential role in care, which starts with CS prevention in primary care, and includes care provided to neonates with CS and their families in hospital environments, and follow-up care to mothers and children. Nursing interventions can empower women for self-care, and consequently, mitigate the occurrence of syphilis and reduce the number of CS cases. Nurses can help handle feelings, minimize stigmatization, support child care, and build parenthood. By understanding the intersubjective aspects involved in this experience, nurses are prepared to rethink their care practice and offer comprehensive and humanized care.

## FINAL CONSIDERATIONS

This study found intense intersubjective aspects that these women experienced and resignified at different moments, from diagnosis of the disease to child follow-up in a specialized service. The diagnosis of syphilis symbolized a shock for the women, who felt responsible for preventing vertical transmission through its proper treatment. On the other hand, the diagnosis of CS represented frustration due to their efforts, in vain, to avoid vertical transmission.

The symbolic meaning of syphilis was aggravated by the fear of finding stigmatizing social attitudes, especially from family members and health professionals, and was reconstructed as women acquired knowledge of the disease and how to heal it. However, guilt was expressed in different moments of having a child affected by CS - enhanced by frustration due to failed treatment and vertical transmission, and by hospitalization and painful procedures required in child care. This study highlights the importance of nurses to provide comprehensive and expanded care, going beyond clinical issues to consider social and emotional needs involved in the experience of these women and their families.

## References

[1] Korenromp EL, Rowley J, Alonso M, Mello MB, Wijesooriya NS, Mahiane SG (2019). Global burden of maternal and congenital syphilis and associated adverse birth outcomes-Estimates for 2016 and progress since 2012. PLOS One.

[2] Wijesooriya NS, Rochat RW, Kamb ML, Turlapati P, Temmerman M, Broutet N (2016). Global burden of maternal and congenital syphilis in 2008 and 2012: a health systems modelling study. Lancet Glob Health.

[3] Padovani C, Oliveira RR, Pelloso SM. (2018). Syphilis in during pregnancy: association of maternal and perinatal characteristics in a region of southern Brazil. Rev Latino-Am Enfermagem.

[4] Oliveira SIM, Oliveira Saraiva COP, Franca DF, Ferreira MA, de Melo Lima LH, de Souza NL (2020). Syphilis notifications and the triggering processes for vertical transmission: a cross-sectional study. Int. J Environ Res Public Health.

[5] Leal MC, Szwarcwald CL, Almeida PVB, Aquino EML, Barreto ML, Barros F (2018). Reproductive, maternal, neonatal and child health in the 30 years since the creation of the Unified Health System (SUS). Ciênc Saúde Colet.

[6] Ministério da Saúde (BR) (2021). Boletim Epidemiológico Sífilis/2021[Internet].

[7] World Health Organization(WHO) (2016). Global health sector strategy on sexually transmitted infections 2016-2021: towards ending STIs-Report[Internet].

[8] Organização das Nações Unidas (ONU) (2015). Transforming our world: the 2030 Agenda for Sustainable Development[Internet].

[9] Liu L, Oza S, Hogan D, Perin J, Rudan I, Lawn JE (2015). Global, regional, and national causes of child mortality in 2000-13, with projections to inform post-2015 priorities: an updated systematic analysis. Lancet.

[10] Veloso FCS, Kassar LML, Oliveira MJC, Lima THB, Bueno NB, Gurgel RQ (2019). Analysis of neonatal mortality risk factors in Brazil: a systematic review and meta-analysis of observational studies. J Pediatr.

[11] Silva JN, Cabral JF, Nascimento VF, Lucietto GC, Cunha Oliveira CB, Silva RA. (2018). Impactos do diagnóstico da infecção sexualmente transmissível na vida da mulher. Enferm Foco.

[12] Guimarães MSF, Santos IMM, Silva LJ, Christoffel MM, Silva LR. (2018). Parenthood of parents of newborns hospitalized due to congenital syphilis in the light of the Transition Theory. Texto Contexto Enferm.

[13] Silva JG, Gomes GC, Ribeiro JP, Nobre CMG, Nörberg PKO, Mota MS. (2019). Congenital syphilis in newborns: repercussions for the mother. Rev Enferm UERJ.

[14] Souza MHT, Beck EQ. (2019). Understanding the congenital syphilis from the maternal look. Rev Enferm UFSM.

[15] Brito APA, Kimura AF. (2018). Transmissão vertical da sífilis: vivência materna durante a hospitalização para diagnóstico e tratamento de seu filho recém-nascido. Rev Paul Enferm [Internet].

[16] Araujo SR, Farias AL, Alcântara DS, Marroni SN, Borges NM, Magalhães CCRGN, Barros LCS, Brito AKL, Costa GD, Bartholomeu LMDO. (2020). Mother’s experience against congenital syphilis occurrence in his children. REAS.

[17] Siqueira MA, Rolim MAD, Ferreira AR, Rocha FAA, Cavalcante MMB. (2017). Sentimentos e conhecimentos de puérperas em face da Sífilis congênita neonatal. Rev Bras Pesq Saúde[Internet].

[18] Lima VC, Mororó RM, Feijão DM, Frota MVV, Martins MA, Ribeiro SM. (2016). Mother’s perception of congenital syphilis in her fetus. Espac Saude [Internet].

[19] Vasconcelos MIO, Oliveira KMC, Magalhães AHR, Guimarães RX, Linhares MSC, Queiroz MV, Albuquerque IMN. (2017). Sífilis na gestação: estratégias e desafios dos enfermeiros da atenção básica para o tratamento simultâneo do casal. Rev Bras Promoc Saúde.

[20] Silva AA, Jardim MJA, Rios CTF, Fonseca LMB, Coimbra LCJ. (2019). Prenatal care of usual-risk pregnant women: potentialities and weaknesses. Rev Enferm UFSM.

[21] Moreira KFA, Oliveira D, Alencar L, Cavalcante D, Pinheiro A, Orfão NJCE. (2017). Profile of notified cases of congenital syphilis. Cogitare Enferm.

[22] Valentim RAM, Caldeira-Silva GJP, da Silva RD (2022). Stochastic Petri net model describing the relationship between reported maternal and congenital syphilis cases in Brazil. BMC Med Inform Decis Mak.

[23] Dalazen CE, Souza AS, Ribeiro CJN, Santos MM, Probst LF, Theobald MR. (2022). Space-time risk cluster and time trends of congenital syphilis in Brazil: an ecological study. Trans Rev Soc Trop Med Hyg.

[24] Charon JM. (2010). Symbolic interactionism: an introduction an interpretation, an integration.

[25] Braun V, Clarke V. (2006). Using thematic analysis in psychology. Qual Res Psychol [Internet].

[26] Tong A, Sainsbury P, Craig J. (2007). Consolidated criteria for reporting qualitative research (COREQ): a 32-item checklist for interviews and focus groups. Int J Qual Health Care.

[27] Minayo MCS. (2017). Amostragem e saturação em pesquisa qualitativa: consensos e controvérsias. Rev Pesqui Qual [Internet].

[28] Cooper JM, Sanchez PJ. (2018). Congenital syphilis. Semin Perinatol.

[29] Ministério da Saúde (BR) (2020). Protocolo Clínico e Diretrizes Terapêuticas para Atenção Integral às Pessoas com Infecções Sexualmente Transmissíveis (IST)[Internet].

[30] Antão C, Rodrigues N, Sousa F, Anes E, Pereira A. (2018). Hospitalização da criança: sentimentos e opiniões dos pais. Rev INFAD Psicol [Internet].

[31] Gonçalves KG, Figueiredo JR, Oliveira SX, Davim RMB, Camboim JCA, Camboim FE. (2017). Criança hospitalizada e equipe de enfermagem: opinião de acompanhantes. Rev Enferm UFPE [Internet].

[32] Feliz MC, Medeiros ARP, Rossoni AM, Tahnus T, Pereira AMVB, Rodrigues CJ. (2016). Adherence to the follow-up of the newborn exposed to syphilis and factors associated with loss to follow-up. Rev Bras Epidemiol.

[33] Domingues CSB, Duarte G, Passos MRL, Sztajnbok DCN, Menezes MLB. (2021). Protocolo Brasileiro para Infecções Sexualmente Transmissíveis 2020: sífilis congênita e criança exposta à sífilis. Consenso Epidemiol Serv Saúde.

